# Epidemic Encephalitis

**Published:** 1919-01

**Authors:** 


**Epidemic Encephalitis.** By ARTHUR HALL, M. D. *The British Medical Journal,* October 26, 1918.

In the early part of 1918 there appeared an epidemic, the nature of which was at first obscure. It was for a time called Botulism, and brought about a closer study of "the alteration of foodstuffs in quantity and quality under war conditions."

Sixteen cases were especially studied and the following observations made : in some cases the onset was sudden; the three cardinal signs of a typical case were lethargy, general asthenia, and cranial nerve palsies.

The clinical picture as a whole is one of immobility, and the face has a mask-like appearance. General asthenia is so extreme that some patients cannot turn over in bed, and can hardly move their arms or legs.

In case 2, a hospital nurse was thought to be suffering from tuberculous meningitis; there was stupor with intervals of delirium, pyrexia, cranial palsies and facial diplegia.

Though there had been no talk of epidemic, Hall connected this case with three others possessing identical signs. Shortly after, patients followed one another so quickly that the existence of a local epidemic became certain.

In most cases there is a distinct interval of from one to eight days between onset and appearance of characteristic symptoms. First there was severe neuralgia on the right side, and a week afterwards vertigo and tinnitus on the left; two days afterwards some stiffness of the right face was the sign observed in case 2.

All sixteen patients treated are alive, but they are not equally well. Eight have made a complete and absolute recovery, six a practically complete recovery, and three an incomplete one. Strabismus is present, so are inequality of pupils and nystagmus. Fever is sometimes severe and prolonged, though not necessarily found in serious attacks, but the most striking feature is lethargy, not true sleep. Many patients become delirious at night, often in a foolish way, suggesting hysteria; some show a type of stupor with katatonia. A woman presented tremors of several days' duration. Often speech was affected at that period. Hall does not dwell on the observations made on cerebro-spinal fluids, bloods, and excreta. He dwells more on the disease as an epidemic. The clinical resemblances with poliomyelitis are evident, though the clearly defined differences stated by Kinnier Wilson make an entirely different disease of encephalitis.

In poliomyelitis the onset tends to be rapid and maximal; the regions affected are mostly those innervated from the spinal cord; the distribution is unilateral. In encephalitis the paralyses are typical of gradual, ingravescent onset; they affect preferably regions innervated by cranial nerves, and are often bilateral; so far, they do not leave permanent residual effects in the area first affected.

Poliomyelitis is considered as an essentially cord disease in its sporadic form, and in its epidemic form it is characterized by the multiformity of sites affected. This argument accentuates the clinical difference of encephalitis in which the uniformity of sites affected is marked.

It is known that a similar epidemic was recorded in 1817 in Vienna, and Netter also had similar cases in Paris. Other chronicles may prove that this encephalitis epidemic is not a new disease.

